# F187. TBSS ANALYSIS OF WHITE MATTER ALTERATIONS IN SCHIZOPHRENIA PATIENTS VS. HEALTHY CONTROLS – RELATION TO AUDITORY VERBAL HALLUCINATIONS

**DOI:** 10.1093/schbul/sby017.718

**Published:** 2018-04-01

**Authors:** Justyna Beresniewicz, Rene Westerhausen, Erik Johnsen, Kenneth Hugdahl, Kristiina Comps

**Affiliations:** 1 University of Bergen; 2 University of Oslo, University of Bergen; 3 University of Bergen, University of Oslo, Haukeland University Hospital

## Abstract

**Background:**

Recent Magnetic Resonance Imaging (MRI) studies on schizophrenia suggest that auditory verbal hallucinations (AVH) might be caused by alterations in connectivity of frontal and temporoparietal language-related areas.3 as well as in connectivity of the default mode network (DMN).1 Therefore, diffusion tensor imaging (DTI) of white matter fiber tracts, subserving anatomical connections between distant and proximal brain regions, could offer complementary information for understanding the anatomical underpinnings of AVH.

**Methods:**

Tract-based spatial statistics (TBSS) allows voxel-wise analysis of multi-subject diffusion data based on fractional anisotropy (FA), assessing microstructural properties of white matter tracts. This study investigates brain white matter tracts in 85 schizophrenia patients and 111 healthy, matched controls using TBSS analysis. Patients were grouped into sub-groups (hallucinating and non-hallucinating) based on the Positive and Negative Syndrome Scale (PANSS), with a cut-off PANSS P3 >= 3 in a 12 months period. Additionally, a comparison between the whole patient sample and controls was performed. The whole-brain analysis was performed with permutation analysis of linear models (PALM).2 Two-tailed t-test was used for group comparison of the patients and controls and a 2 x 2 factorial design was used for hemispheric comparison between patients and controls.

**Results:**

TBSS results shows significantly lower fractional anisotropy in the right inferior longitudinal fasciculus in schizophrenia patients in comparison to healthy controls (FDR correction p < 0.05). However, after subtracting non-hallucinating patients from the group this effect was no longer present. Hemispheric comparison between patients and healthy controls revealed wide- spread FA reduction in several white matter pathways such as: the corpus callosum (genu and body), cingulum cortex, inferior longitudinal fasciculus, superior corona radiata, anterior thalamic radiation (FDR correction p < 0.05). This effect was also present after excluding the non-hallucinating patients from the sample.

**Discussion:**

The present findings indicate reduced white matter integrity in schizophrenia patients compared with controls in the inferior longitudinal fasciculus. However, this observation is most likely not related to hallucination proneness since it was not present when comparing the hallucinating patient subgroup with the control group. Hemispheric comparison between patients and controls in both the whole data sample as well as hallucinating group showed significant differences in several white matter pathways. This could indicate that schizophrenia patients (with or without AVH) have altered FA differences between the hemispheres. More research is needed to further understand the implications of these findings.

**References:**

1. Kim, D., Manoach, D., Mathalon, D., Turner, J., Mannell, M., Brown, G., . . . Calhoun, V. (2009, November). Dysregulation of working memory and default-mode networks in schizophrenia using independent component analysis, an fBIRN and MCIC study. Human Brain Mapping, 3795–811.

2. Winkler, A. M., Ridgway, G. R., Webster, M. A., Smith, S. M., & Nichols, T. E. (2014). Permutation inference for the general linear model. Elsevier, 381–397.

3. Zhang, L., Li, B., Wang, H., Li, L., Liao, Q., Liu, Y., . . . Tan, Q. (2017). Decreased middle temporal gyrus connectivity in the language network in schizophrenia patients with auditory verbal hallucinations. Neurscience Letters, 177–182.

